# Targeted regulation of miR-154-5p/Cullin2 pathway by hsa_circ_TRIM22 in promoting human papillomavirus 16 positive cervical cancer progression

**DOI:** 10.7150/jca.92631

**Published:** 2024-02-24

**Authors:** Weihong Zhao, Songquan Wen, Xiuting Wang, Jingfang Wang, Lili Zhang, Tong Wang

**Affiliations:** 1 Department of Obstetrics and Gynecology, The Second Hospital of Shanxi Medical University, Taiyuan, Shanxi Province, China.; 2 Department of Biochemistry and Molecular Biology, Basic Medical College, Shanxi Medical University, Taiyuan, Shanxi Province, China.; 3 Department of Health Statistics, School of Public Health, Shanxi Medical University, Taiyuan, Shanxi Province, China.

**Keywords:** Cervical cancer, HPV16, hsa_circ_TRIM22, miR-154-5p, CUL2

## Abstract

**Background.** Tripartite motif-containing 22 (TRIM22) is characterized by a canonical RING domain with ubiquitin E3 ligase activity and is closely associated with tumorigenesis. As a product of TRIM22 transcription, whether hsa_circ_TRIM22 has a function of regulating tumorigenesis is unclear. Thus, we aimed to explore the role and mechanism of hsa_circ_TRIM22 in human papillomavirus (HPV) 16 positive cervical cancer (CC).

**Methods.** We collected HPV16-positive cervical tissues including chronic cervicitis, high-grade squamous intraepithelial lesions (HSIL), low-grade squamous intraepithelial lesions (LSIL), and CC, and along with CC cell lines to detect the hsa_circ_TRIM22 level using real-time fluorescence quantitative polymerase chain reaction (RT-qPCR). Hsa_circ_TRIM22 was silenced using specific short hairpin ribonucleic acid (shRNA) in CC cell lines and functional assays were performed thereafter. Mechanistically, the targeting and regulatory relationship between hsa_circ_TRIM22 and miR-154-5p were confirmed using the luciferase report assay and rescue experiments.

**Results.** We found hsa_circ_TRIM22 expression level was significantly higher in CC cells and tissues. Further, hsa_circ_TRIM22 knockdown inhibited migration, proliferation, invasiveness, enhanced apoptosis, and slowed the cell cycle. Mechanistically, hsa_circ_TRIM22 could bind miR-154-5p and prevent miR-154-5p from reducing the levels of Cullin2 (CUL2). Notably, the application of miR-154-5p inhibitor significantly rescued hsa_circ_TRIM22-mediated tumorigenesis.

**Conclusions.** Our observations suggest hsa_circ_TRIM22 is upregulated in HPV16-positive CC and promotes CC progression by regulating the miR-154-5p/CUL2 axis, thereby serving as a promising candidate for diagnosis and treatments of CC.

## Introduction

Cervical cancer (CC) is the most common gynecological malignancy among women worldwide. It is characterized by high rates of morbidity and mortality, often due to late detection and lack of access to affordable healthcare. Oncogenic human papillomavirus (HPV) infection is a major causative factor, with HPV16 infection accounting for >50% of CC 1. The conventional mechanism involves the degradation of the tumor suppressor retinoblastoma protein (pRb) by the E7 protein of HPV16. HPV16 E7 binds to the Cullin-RING ubiquitin E3 ligase Cullin2 (CUL2) and mediates pRb ubiquitination and degradation, resulting in the malignant transformation of cells 2. Therefore, regulating CUL2 expression is crucial to inhibit the HPV16 E7-pRb oncogenic pathway.

The microRNA, miR-154-5p, inhibits CC development by targeting and silencing CUL2 3,4; however, its upstream regulatory mechanisms remain unknown. As a novel type of endogenous non-coding ribonucleic acid, circRNA has a highly conserved, stable, and specific closed circular structure and competitively binds to microRNA (miRNA), thus inhibiting the degradability of their corresponding messenger RNA (mRNA). Therefore, circRNA regulates the expression of oncogenes and tumor suppressor genes in tumors and plays a critical role in the development of tumors 5,6. In light of this, we had previously employed RNA microarray analysis and found that hsa_circ_0000276 has the highest ability (score = 167, energy = -27.76) for binding to miR-154-5p 7. Furthermore, hsa_circ_0000276 is on human chromosome 11 at chr11:5711031-5717503 with a total length of 6472 bp. Its parent transcript originates from tripartite motif (TRIM)-containing 22 (TRIM22). TRIM22 is characterized by a canonical RING domain with ubiquitin E3 ligase activity and is closely associated with tumorigenesis 8. The expression and role of TRIM22 vary in different tumor tissues. Its expression decreased in breast cancer 9, osteosarcoma 10, and gastric cancer 11, and inhibited the progression of these tumors. However, TRIM22 is significantly upregulated in Ishikawa endometrial cancer cells 12, glioblastoma cells 13, and colon cancer cells 14, where it acts as an oncogene. However, as a product of TRIM22 transcription, whether hsa_circ_0000276 (hsa_circ_TRIM22) has a function of regulating tumorigenesis is unclear. Thus, our study aimed to explore the role of hsa_circ_TRIM22 in CC and revealed the molecular pathogenesis by which it targets the miR-154-5p/CUL2 pathway to regulate CC tumorigenesis.

## Materials & methods

### Tissue samples

Fifty-six samples were collected, including 10, 19, 11, and 16 of chronic cervicitis, LSIL, HSIL, and CC, respectively, in subjects who underwent colposcopic examination at the Second Hospital of Shanxi Medical University between January and June 2022. All subjects were positive for a single HPV16 infection. The average age of women with chronic cervicitis, LSIL, HSIL, and CC was 44.10±9.78, 43.58±7.14, 42.00±8.35, and 49.69±6.760 years, respectively. All CC tissues were cervical squamous cell carcinoma tissues. According to the 2009 International Federation of Gynecology and Obstetrics (FIGO) staging standard, 5 cases of stage Ia, 6 cases of stage Ib, and 5 cases of stage IIa cancer were identified. According to pathological classification, 2 cases of well-differentiated, 9 cases of moderately differentiated, and 5 cases of poorly differentiated cancers were identified. Two experienced clinical pathologists independently diagnosed tissue samples. Inclusion criteria: (1) married women aged ≤65; (2) local resident for at least one year; and (3) written informed consent. Anyone with any of the following was excluded: (1) pregnancy; (2) hysterectomy history; (3) cervical or vaginal lesion treatment history; and (4) other malignancies. The study was approved by the Ethics Committee of the Second Hospital of Shanxi Medical University [IRB no. (2019) YX (280)] and conducted in compliance with its regulations. Written informed consent was obtained from the participants.

### Cell culture

SiHa and HCerEpiC cells were acquired from the Cell Center of Shanghai Institutes for Biological Sciences (Shanghai, China), which were retained in high-glucose Dulbecco's Modified Eagle Medium (DMEM) (Seven, Beijing, China) supplemented with 10% fetal bovine serum (FBS) (sangon, Shanghai, China). CaSki cells were cultured in Roswell Park Memorial Institute 1640 medium (Seven, Beijing, China) supplemented with 10% FBS. These cells were incubated at 5% CO_2_, 37 °C, and 95% humidity, and the media were changed every alternate day.

### Cell transfection

A short hairpin ribonucleic acid (shRNA) expression vector and miR-154-5p inhibitor oligonucleotides (GenePharma, Shanghai, China) were designed based on the sequences of hsa_circ_TRIM22 and miR-154-5p, respectively. CC cells were cultured in a 6-well plate and transfected with plasmid or RNA oligos (100 nM) using Lipofectamine 2000 (Invitrogen, CA, USA) when they reached 70% confluence. The medium was changed after 4-6 h, and the cells were harvested for real-time fluorescence quantitative polymerase chain reaction (RT-PCR) after 24 h (hsa_circ_TRIM22 knockdown) or 48 h (rescue experiment). The sequences are shown as follows: hsa_circ_TRIM22 shRNA (sh-hsa_circ_TRIM22): GCCAGAGGGCTGGTCACCT, shRNA negative control (sh-NC): GTTCTCCGAACGTGTCACGT.

### Ribonucleic acid extraction and RT-qPCR

The RNeasy Mini Kit (Qiagen, Wellington, Germany) was used to extract total RNA from collected tissues and cells. Complementary deoxyribonucleic acids of RNAs (circRNA, miRNA, and mRNA) were synthesized using the PrimeScript^TM^ Reverse Transcriptase reagent kit (TAKARA, Dalian, China). Thereafter, RT-qPCR was performed using the TB Green kit (TAKARA, Dalian, China) and the Applied Biosystems 7500 Fast Real-Time PCR System (Applied Biosystems, Inc. Carlsbad, CA, USA). U6 and glyceraldehyde 3-phosphate dehydrogenase (GAPDH) constituted the internal controls. The primer sequences used in this study were shown in Table 1.

### Cell Counting Kit-8 analysis

The Cell Counting Kit-8 (CCK-8) (Absin, Shanghai, China) was used to determine the CC cell proliferation rate. Transfected cells were inoculated into a 96-well plate at a density of 3000 cells/well. After 24 h in culture, 100 μL of serum-free medium containing 10% CCK-8 reagent was added to each well. A plate reader (AutoBio, Zhengzhou, China) was used to measure the absorbance at 450 nm, 2 h after incubation.

### Scratch wound healing assay

When the cells in the 6-well plates reached approximately 90% confluence at 24 h post-transfection, a 200-μL pipette tip was used to make a straight-line scratch. The cells were washed twice in phosphate-buffered saline (PBS) (Seven, Beijing, China), serum-free DMEM was added, and the cells were examined under a microscope; this was designated time “0 h.” The cells were placed in an incubator for 24 h and subsequently observed and imaged; this was designated time “24 h.”

### Transwell invasion assay

A Transwell chamber (Corning, New York, USA) was used to perform the invasion assay on CC cells. Initially, 40 μL of diluted Matrigel (Corning, New York, USA) was added to each chamber, which was thereafter transferred to a 24-well plate and placed in an incubator for 3 h. Each chamber was inoculated with 1 × 10^5^ cells. The cells were stained with crystal violet after 48 h and imaged for analysis.

### Cell cycle analysis

The NovoCyte 3130 flow cytometer (Agilent, San Diego, CA, USA) was used to determine the cell cycle status of transfected CC cells. A cell cycle detection kit (Yeasen, Shanghai, China) was used according to the manufacturer's instructions. In total, 1 × 10^6^ cells were collected from each sample, fixed in 70% ethanol at 4 °C for at least 2 h, and washed in PBS. Subsequently, 400 μL of propidium iodide (PI) was added to the suspension for 30 min, followed by detection with NovoCyte flow cytometer (ACEA Pharma, Hangzhou, China).

### Apoptosis assays

The FC500 flow cytometer (Beckman Coulter, Los Angeles, CA, USA) was used to detect the apoptosis of transfected CC cells. The Annexin V-FITC/PI double-dye apoptosis detection kit (Seven, Beijing, China) was used according to the manufacturer's instructions. Overall, 1-5 × 10^5^ cells were collected from each sample. Flow cytometry was performed after staining for 15 min away from light.

### Dual-luciferase reporter assay

Wild-type (WT) and mutant (MUT) hsa_circ_TRIM22 gene fragments were designed, synthesized, and cloned into the GenePharma miRGLO plasmid (Promega, WI, USA). Thereafter, 293T cells were inoculated in 12-well plates, and the GP-transfect-Mate transfection reagent (GenePharma, Shanghai, China) was used to co-transfect the luciferase reporter vector with a miR-154-5p mimic. The medium was changed, 5 h post-incubation. After incubating for 24 h, as per the instructions provided with the Dual-Luciferase Assay Kit (Promega, WI, USA), the culture medium was discarded, the cells were washed twice with PBS, and cell lysis buffer was added to lyse the cells. Firefly luciferase reaction buffer and the corresponding substrate were mixed thoroughly to detect firefly luciferase activity. Subsequently, the *Renilla* luciferase reaction buffer and corresponding substrate were added and mixed thoroughly to detect *Renilla* luciferase activity.

### Western blotting

Total proteins were extracted from cells using radioimmunoprecipitation assay lysis buffer (Boster, Wuhan, China). Protein concentrations were determined using the bicinchoninic acid assay (Boster, Wuhan, China). Protein (30 μg) was separated using 8% sodium dodecyl sulfate-polyacrylamide gel electrophoresis, and later electroblotted onto a polyvinylidene fluoride membrane. The membranes were blocked with 5% skimmed milk at 37 °C for 2 h and incubated overnight at 4 ℃with CUL2 antibody (Invitrogen, CA, USA) or β-actin antibody (CST, MA, USA). The following day, the membranes were incubated with anti-HRP rabbit antibody at 37 ℃ for 1 h and treated with an ultra-high sensitivity enhanced chemiluminescence reagent (Boster, Wuhan, China).

### Statistical analysis

GraphPad Prism (v. 8.0) and IBM SPSS 26.0 (Statistical Package for the Social Sciences, IBM Corp., Armonk, NY, USA) were used for statistical analysis. Data are expressed as mean (X) ± standard deviation (S) for variables with normal distribution. Differences among three or more groups of data were compared using analysis of variance. The Mann-Whitney and independent samples t-tests were used to compare differences between two groups. Differences with *P*< 0.05 were considered significant.

## Results

### Hsa_circ_TRIM22 expression in different cervical tissues and cells

**Figure 1:**
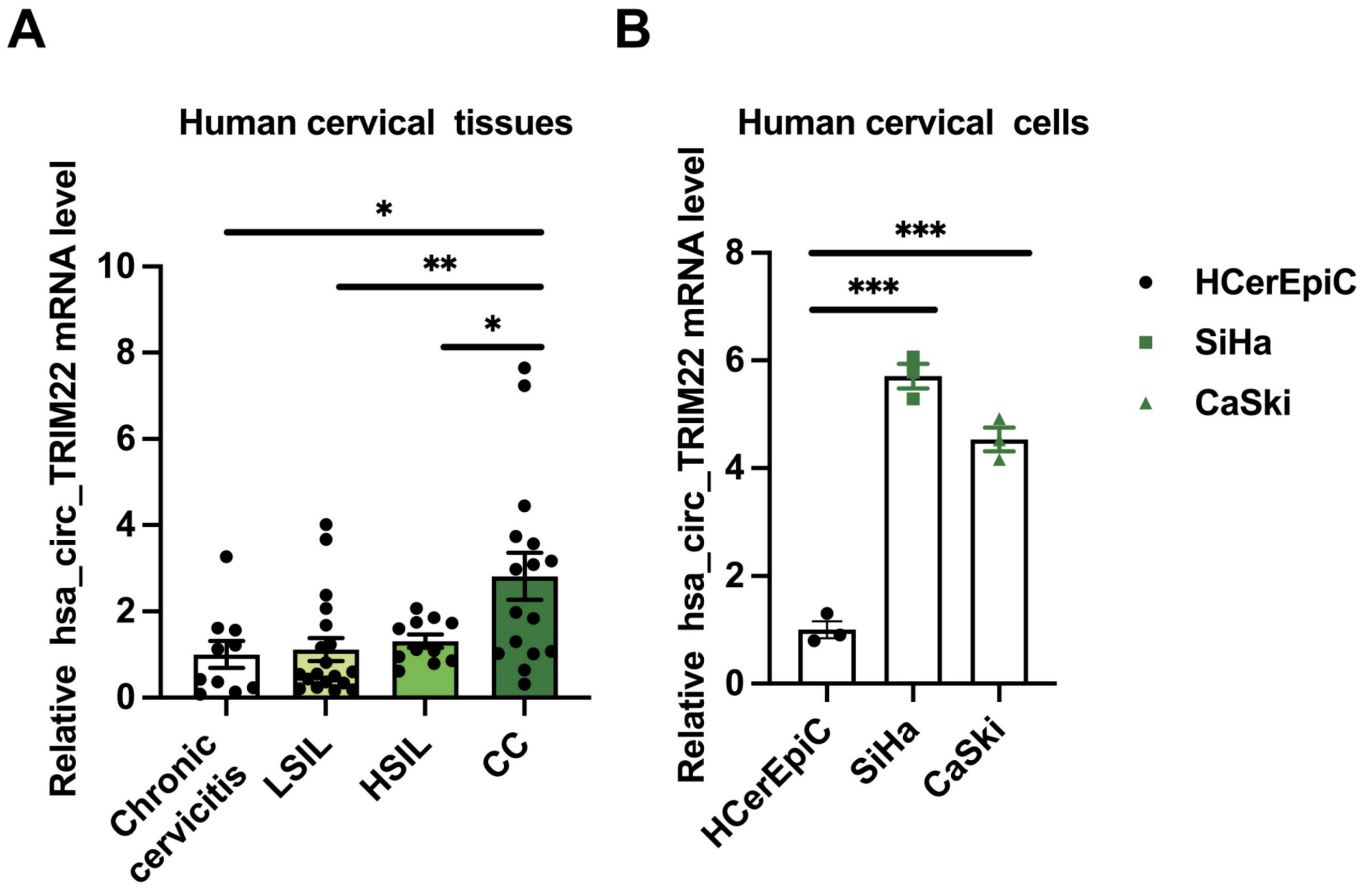
Expression of hsa_circ_TRIM22 in cervical cancer tissues and cells. (A) Results of RT-qPCR analysis revealing the expression of hsa_circ_TRIM22 in cervical cancer tissues in comparison with chronic cervicitis and SIL tissues; *, *P*<0.05; **, *P*<0.01. (B) Results of RT-qPCR analysis revealing the expression of hsa_circ_TRIM22 in cervical cancer cells in comparison with normal human cervical epithelial cells; ***, *P*<0.001.

RT-qPCR examination of cervical tissues and cells revealed differences in the expression levels of hsa_circ_TRIM22 in the chronic cervicitis, LSIL, HSIL, and CC groups. Further pairwise comparisons indicated that hsa_circ_TRIM22 expression in the CC tissues was higher than that in the chronic cervicitis, LSIL, and HSIL groups (Fig. 1A). The relative levels of hsa_circ_TRIM22 expression differed among HCerEpiC, SiHa, and CaSki cells. Further pairwise comparisons demonstrated that hsa_circ_TRIM22 expression was also higher in the SiHa and CaSki cells than in the HCerEpiC cell lines (Fig. 1B).

### Changes in the biological characteristics of CC cells after silencing hsa_circ_TRIM22

**Figure 2:**
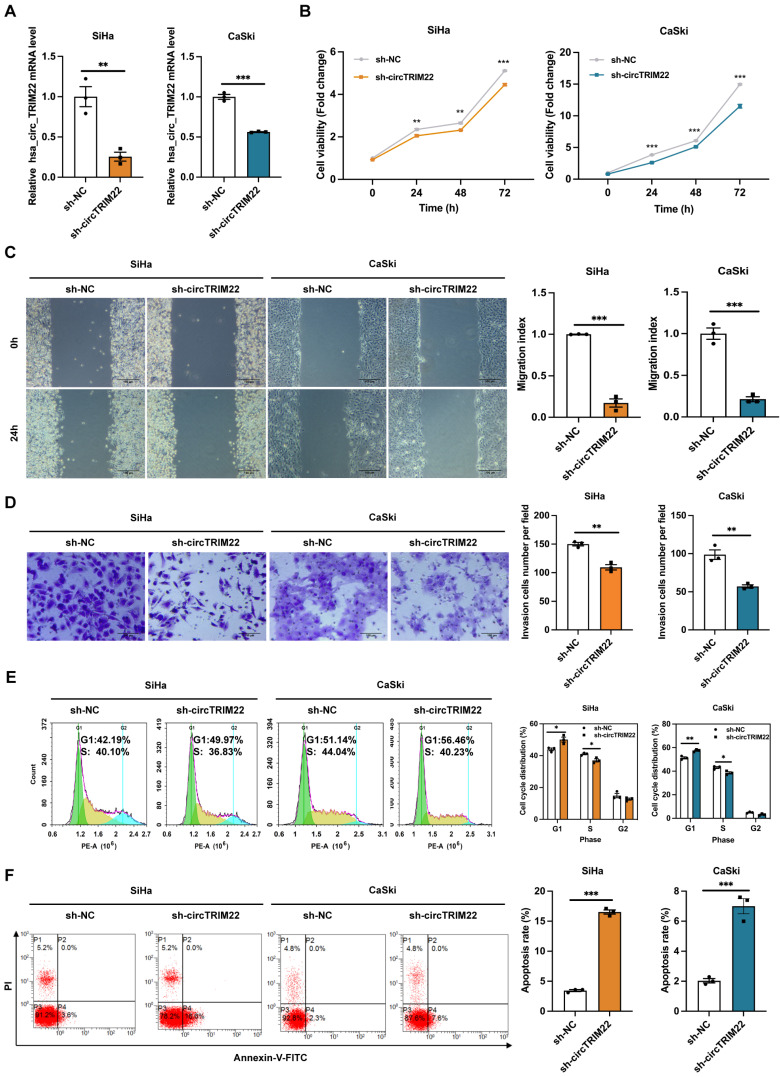
Effect of hsa_circ_TRIM22 shRNA on the malignant biological behavior of cervical cancer cells. (A) Results of RT-qPCR analysis revealing hsa_circ_TRIM22 expression in SiHa and CaSki transfected with shRNA; **, *P*<0.01; ***, *P*<0.001. (B) Growth curves of SiHa and CaSki transfected with shRNA, as evaluated using CCK8 assays; **, *P*<0.01; ***, *P*<0.001. (C) Cell migration capacities, as detected using scratch healing assays after transfection; Scale bar, 100 μm; ***, *P*<0.001. (D) Cell invasion abilities, as determined using Transwell assays after transfection; Scale bar, 100 μm; .**, *P*<0.01. (E) Cell progression, as analyzed using flow cytometry after transfection; *, *P*<0.05; **, *P*<0.01. (F) Cell apoptosis, as assessed using flow cytometry after transfection; ***, *P*<0.001.

To further investigate the role of hsa_circ_TRIM22 in CC progression, shRNAs with hsa_circ_TRIM22 knockdown were designed, and the transfection efficiency of these shRNAs was confirmed. Furthermore, hsa_circ_TRIM22 expression in CC cells in the sh-hsa_circ_TRIM22 group reduced when compared with the sh-NC group (Fig. 2A). Subsequently, we evaluated the effect of hsa_circ_TRIM22 on the biological behavior of CC. As shown using the CCK8 assay, the cell viability of CC cells was significantly inhibited at 24, 48 and 72 h after transfection with hsa_circ_TRIM22 shRNA compared with the sh-NC group (Fig.2B). We assessed the migration capabilities of CC cells by the scratch assay. As shown in Fig. 2C, hsa_circ_TRIM22 knockdown restrained the migration of CC cells in comparison to the control group. Similar results were also obtained in the Transwell assays. Our data revealed that hsa_circ_TRIM22 knockdown significantly downregulated the cell invasion abilities in CC cells (Fig. 2D). Cell cycle distribution was measured by flow cytometry using the PI staining method. As shown in Fig. 2E, the proportion of cells in the G1 phase was increased in the sh-hsa_circ_TRIM2 group compared to the sh-NC group, which was accompanied by a marginal decrease in the proportions of cells in S phase. We also used FITC/PI double staining assay to detect cell apoptosis by flow cytometry. Results showed that the apoptotic rate was increased after knocking down hsa_circ_TRIM22 in CC cells compared to sh-NC group (Fig.2F).

### Validation of targeted regulation of miR-154-5p expression by hsa_circ_TRIM22

**Figure 3:**
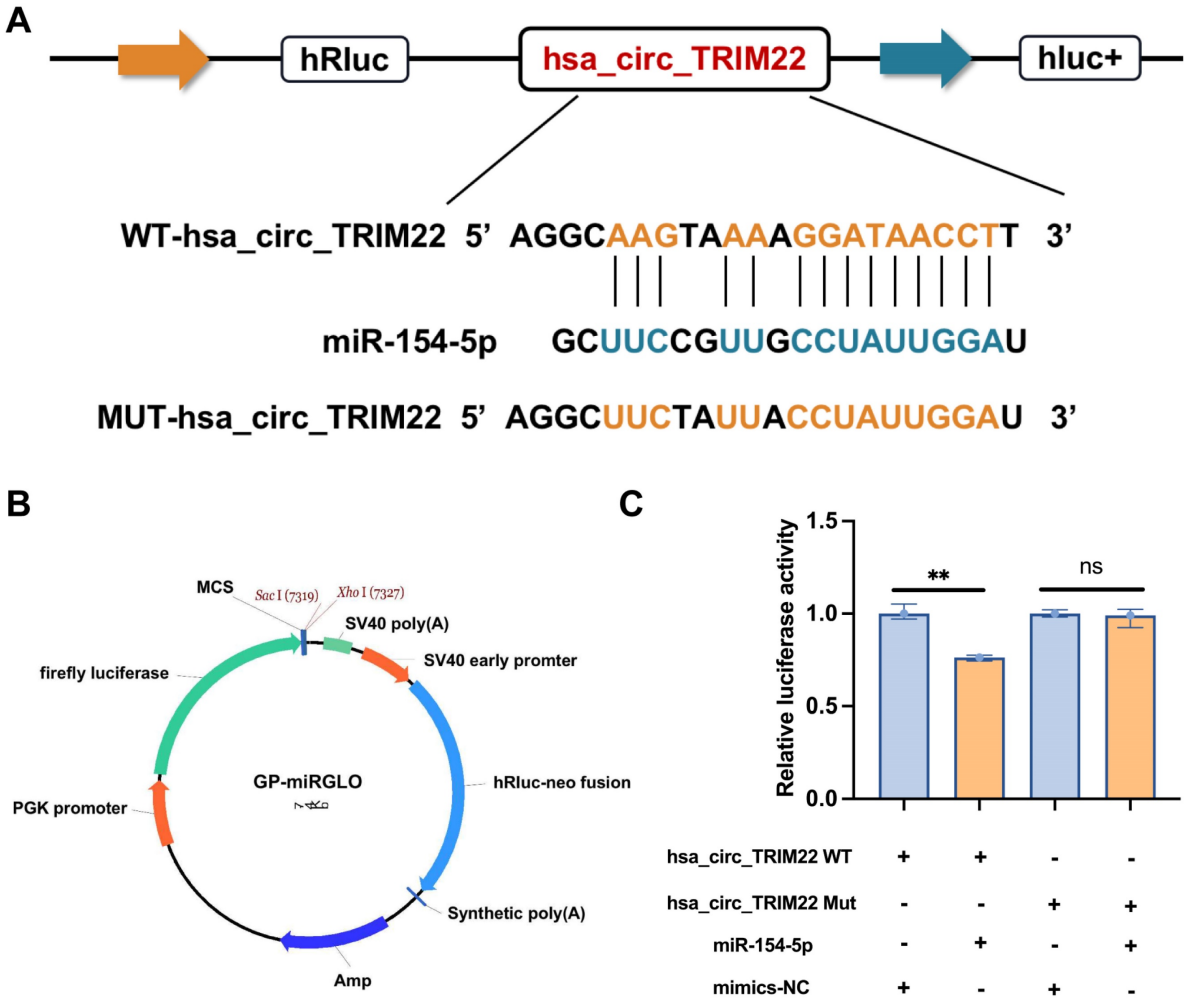
Hsa_circ_TRIM22 directly binds to miR-154-5p. (A) Schematic illustration of hsa_circ_TRIM22 WT and hsa_circ_TRIM22 MUT luciferase reporter vectors. (B) Map information of GP-miRGLO plasmid. (C) Relative luciferase activities were detected in 293T cells after transfection with hsa_circ_TRIM WT or hsa_circ_TRIM22 MUT and miR-154-5p mimics or mimics-NC, respectively; **, *P*<0.01; ns, no significance.

Bioinformatics predictions indicated that hsa_circ_TRIM22 has a consecutive binding site to the miR-154-5p nucleotide sequence. The wild-type (WT) and mutant dual-luciferase reporter plasmids of hsa_circ_TRIM22 were constructed with GP-miRGLO plasmid (Figs. 3A-B). The data revealed that the luciferase activity of cells in the hsa_circ_TRIM22 WT group was inhibited by miR-154-5p binding; the difference was significant. No significant difference was observed in the luciferase activity of cells in the hsa_circ_TRIM22 MUT group when compared to the control group (Fig. 3C). This implied that hsa_circ_TRIM22 directly regulated miR-154-5p.

### Changes in miR-154-5p and CUL2 expression in CC cells after silencing hsa_circ_TRIM22

**Figure 4:**
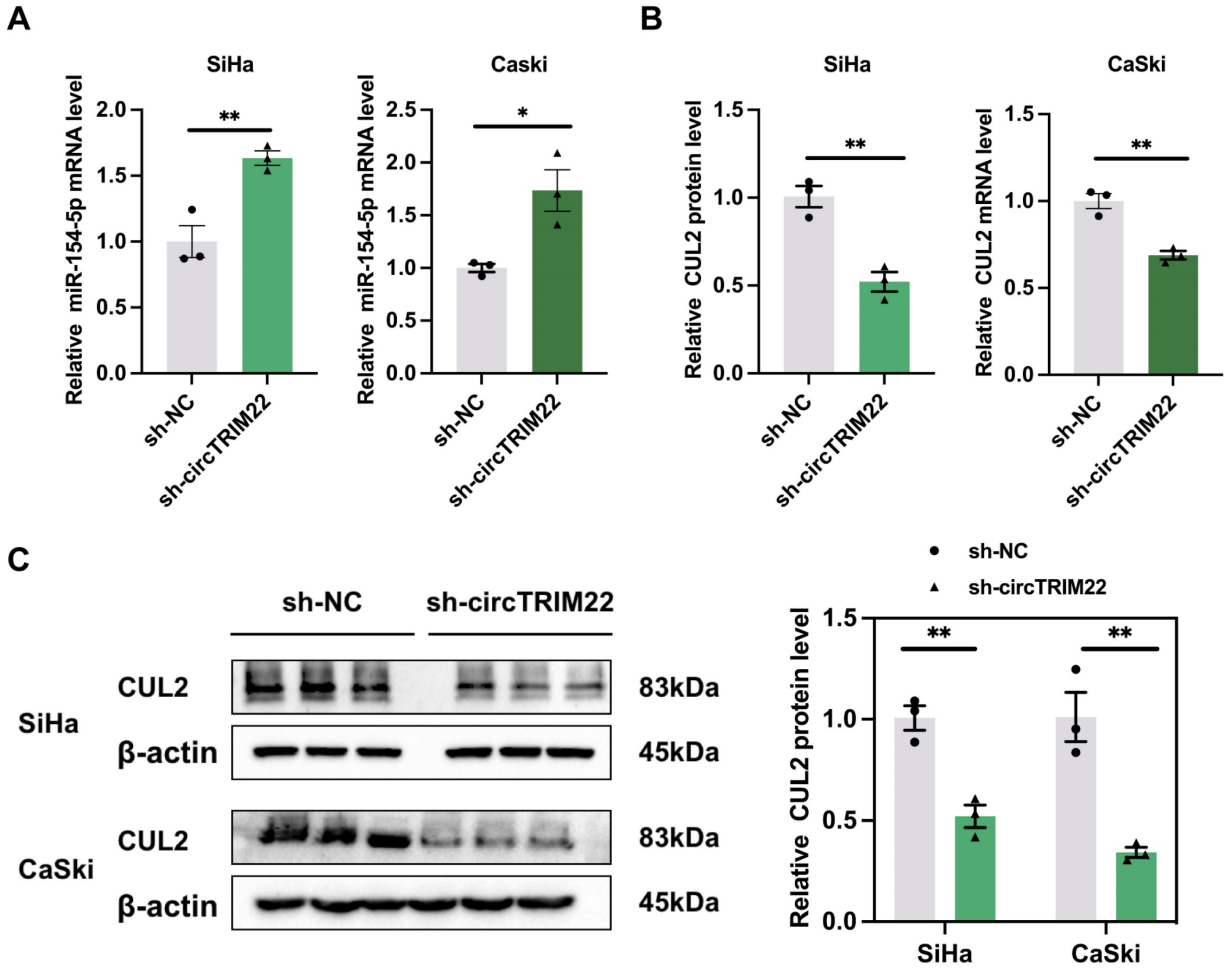
Impact of Hsa_circ_TRIM22 on miR-154-5p/CUL2 expression. (A) Relative expression of miR-154-5p, as detected using RT-qPCR after transfected with hsa_circ_TRIM22 shRNA; *, *P*<0.05; **, *P*<0.01. (B) Relative expression of CUL2 mRNA, as detected using RT-qPCR after transfected with hsa_circ_TRIM22 shRNA; **, *P*<0.01. (C) Relative expression of CUL2 protein, as detected using western blotting after transfected with hsa_circ_TRIM22 shRNA; **, *P*<0.01.

Hsa_circ_TRIM22 exerts targeted regulation of miR-154-5p expression. Our results showed that miR-154-5p expression increased in CC cells after transfection with sh-hsa_circ_TRIM22 than sh-NC (Fig. 4A). Our previous studies confirmed that CUL2 is a downstream target regulated by miR-154-5p; therefore, CUL2 expression was also determined. We also found that both CUL2 mRNA and protein expression were decreased in CC cells after transfection with sh-hsa_circ_TRIM22 than sh-NC (Figs. 4B-C).

### Changes in CUL2 protein expression and biological behaviors of CC cells after silencing hsa_circ_TRIM22 and miR-154-5p downregulation

**Figure 5:**
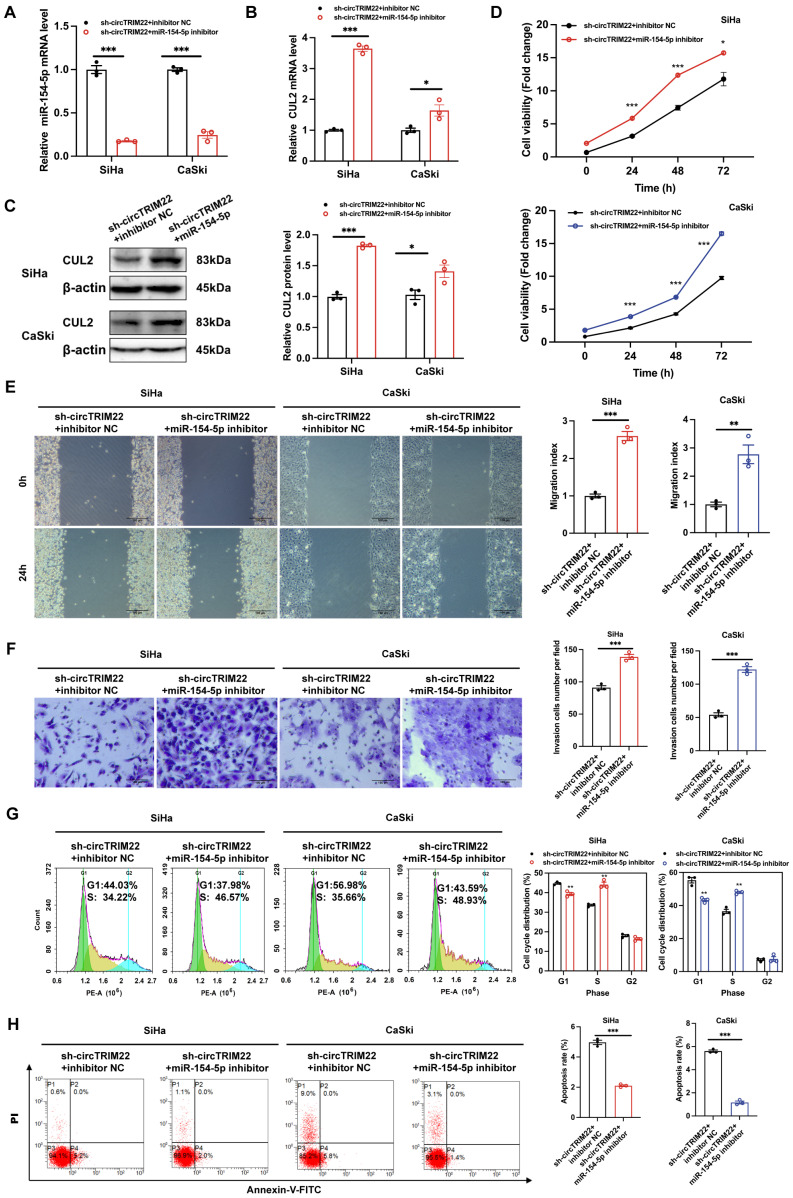
Influences of hsa_circ_TRIM22 shRNA on the malignant biological behavior of cervical cancer cells through the miR-154-5p/CUL2 axis. (A) Relative expression of miR-154-5p, as detected using RT-qPCR in CC cells with the transfection of both sh-hsa_circ_TRIM22 and miR-154-5p inhibitor; ***, *P*<0.001. (B-C) Relative expression of CUL2 mRNA and protein, as detected using RT-qPCR and western blotting in CC cells with the transfection of both sh hsa_circ_TRIM22 and miR-154-5p inhibitor; *, *P*<0.05; ***, *P*<0.001. (D) Growth curves of CC cells in the indicated groups, as evaluated using CCK8 assays; *, *P*<0.05; ***, *P*<0.001. (E) Cell migration capacities, as detected using scratch healing assays in the indicated groups; **, *P*<0.01; ***, *P*<0.001. (F) Cell invasion abilities, as determined using Transwell migration assay in the indicated groups; ***, *P*<0.001. (G) Cell progression, as revealed using flow cytometry in the indicated groups; **, *P*<0.01. (H) Cell apoptosis, as revealed using flow cytometry in the indicated groups; ***, *P*<0.001.

To determine whether hsa_circ_TRIM22 functions via the miR-154-5p/CUL2 pathway, functional recovery experiments were performed using a miR-154-5p inhibitor. The experiment was divided into sh-hsa_circ_TRIM22 + inhibitor NC and sh-hsa_circ_TRIM22 + miR-154-5p inhibitor groups. RT-qPCR revealed that miR-154-5p expression in CC cells in the sh-hsa_circ_TRIM22 + miR-154-5p inhibitor group decreased when compared to the sh-hsa_circ_TRIM22 + inhibitor NC group (Fig. 5A). RT-qPCR and western blotting revealed that CUL2 expression increased after silencing hsa_circ_TRIM22 and miR-154-5p downregulation (Figs. 5B-C). Then, we performed functional experiments in two groups. As shown by CCK8 assay, the cell viability of CC cells was significantly promoted at 24 h, 48 h and 72 h after transfection with hsa_circ_TRIM22 shRNA and miR-154-5p inhibitor compared with the inhibitor NC group (Fig. 5D). Similarly, the migration and invasiveness of CC cells in the sh-hsa_circ_TRIM22 + miR-154-5p inhibitor group increased compared to the control group with a significant difference (Fig. 5E and 5F). The proportion of cells in the G1 phase was decreased, but the proportion of cells in the S phase was increased in the sh-hsa_circ_TRIM22+miR-154-5p inhibitor group compared to hsa_circ_TRIM22+inhibitor NC group (Fig. 5G). Finally, our results showed that the apoptotic rate was decreased after inhibiting the miR-154-5p in sh-hsa_circ_TRIM22 CC cells compared to inhibitor NC group (Fig. 5H).

## Discussion

CC development and progression involve multiple genes, factors, and stages 15. Studying the pathways of HPV infection can improve our understanding of CC pathogenesis and provide novel targeted therapy as a treatment option. Ubiquitination is a major pathway for post-translational regulation of proteins. CUL2, an important member of the Cullin-RING ubiquitin E3 ligase family, promotes tumor angiogenesis, regulates cell motility, induces tumor immune escape, and regulates cell proliferation 16. CUL2 is critical in CC development and progression owing to its involvement in the canonical HPV E7-pRb carcinogenesis pathway. We previously demonstrated that miR-154-5p competitively binds to CUL2 mRNA, thereby reducing CUL2 protein expression and inhibiting CC growth 3. To further investigate the upstream regulatory mechanisms of miR-154-5p, bioinformatics prediction software indicated the presence of a binding site in hsa_circ_TRIM22 for the nucleotide sequence of miR-154-5p 7. Further, we performed dual-luciferase reporter gene assays and confirmed a direct regulatory role of hsa_circ_TRIM22 on miR-154-5p. However, no studies on the role of hsa_circ_TRIM22 in diseases have been reported.

The parent transcript of hsa_circ_TRIM22 originates from TRIM22. Because circRNAs have been proposed to exhibit similar characteristics with their coding genes, such as histone modification and DNA methylation activity, maternal effects, and post-transcriptional regulation 17. Given these characteristics, it is worth exploring whether hsa_circ_TRIM22 could also be a novel tumor molecule. Thus, we first confirmed hsa_circ_TRIM22 expression in cervical tissues and cells using RT-qPCR. Hsa_circ_TRIM22 expression was significantly upregulated in CC tissues and cells than in normal tissues and cells. Thereafter, we performed a loss-of-function assay on HPV16-positive CC cells and observed that silencing hsa_circ_TRIM22 *in vitro* inhibited CC cell proliferation, migration, and invasiveness, slowed cell cycle progression, and promoted apoptosis. Our results suggested that hsa_circ_TRIM22 plays an oncogenic role in HPV16-positive CC.

CircRNA, as a recently discovered type of regulatory molecule, is closely related to tumor development. Novel evidence reveals that dysregulated circRNAs influence cancer growth and progression by promoting cell proliferation, cell invasion and migration, preventing apoptosis, and inducing angiogenesis 18. The mechanisms by which circRNAs exert these effects include sponging miRNAs, interacting with RNA-binding proteins or encoding tumor-associated functional peptides 19-22. However, they are currently best known as ceRNAs in tumors as they possess high-affinity miRNA-binding sites. The ceRNA mechanism of circRNAs in the progression of CC have been studied recently 22-24. For example, circRNA hsa_circ_0043280 has demonstrated tumor suppressor activity that inhibits the growth and metastasis of CC tumors via the miR-203a-3p/PAQR3 axis 22. However, circ0001955 promotes CC tumorigenesis and metastasis via the miR-188-3p/NCAPG2 axis 25. In our study, based on the bioinformatics analyses and dual-luciferase reporter results, we found miR-154-5p was upregulated, and CUL2 was downregulated after silencing hsa_circ_TRIM22 in CC cells, suggesting that targeted miR-154-5p silencing by hsa_circ_TRIM22 regulates CUL2 expression in CC cells. Further functional recovery experiments on co-transfected CC cells revealed that miR-154-5p downregulation upregulated mRNA and CUL2 protein expression, while reversing the effects of hsa_circ_TRIM22 silencing on migration, proliferation, invasiveness, cell cycle, and apoptosis of CC cells. Therefore, we concluded that hsa_circ_TRIM22 acts as a ceRNA to promote CC progression via the miR-154-5p/CUL2 axis.

In summary, we found that hsa_circ_TRIM22 was overexpressed in CC. Additionally, we demonstrated that hsa_circ_TRIM22 promoted the proliferation and migration of HPV16-positive CC cells through miR-154-5p/CUL2 axis. Owing to their stable circular structure, resistance to degradation by ribonucleases, and ability to be secreted via extracellular vesicles and exosomes, circRNAs have been detected in blood and saliva, serving as targets for diagnosis and targets for treating diseases. We believe that circRNAs can provide new insights and potential therapeutic targets for diagnosis and treatment of HPV16-positive CC. However, our current study has some limitations. Firstly, our results need to be verified through *in vivo* experiments. Secondly, because cervical squamous cell carcinoma accounts for 80-85% of all types of CC and HPV16 is the most frequent type, we chose two HPV16-positive cancer cells to investigate. But the role of hsa_circ_TRIM22 in other types of CC is unknown and needs to be further improved.

## Conclusions

Taken together, our findings mainly confirmed that hsa_circ_TRIM22 is an oncogenic circRNA implicated in CC occurrence, and it silenced miR-154-5p expression, which results in the upregulation of Cullin2, and thus CC progression. We believed that hsa_circ_TRIM22/miR-154-5p/Cullin2 axis is a promising diagnostic biomarker and therapeutic target in CC.

## Funding

This research was supported by the Outstanding Youth Fund Project of Shanxi Province (No. 201901D211506), Research Project Supported by Shanxi Scholarship Council of China (No. 2022-195), National Natural Science Foundation of China (No. 81702583), China Postdoctoral Science Foundation (No. 2019M651072), Shanxi Province Key National Science and Technology Cooperation Projects (No.202104041101006), Natural Science Foundation of Shanxi Province (No. 201901D111364), and Shanxi Graduate Education Project (No. 2022-076).

## Figures and Tables

**Table 1:** The sequences of primers and oligonucleotides used in this study.

Primer / Oligonucleotide	Sequence (5'-3')
H-U6 F	CGCTTCGGCAGCACATATC
H-U6 R	TTCACGAATTTGCGTGTCATC
miR-154-5p-F	TCTGCCGTAGGTTATCCGTG
miR-154-5p-R	CAGAGCAGGGTCCGAGGTA
GAPDH-F	CAGGAGGCATTGCTGATGAT
GAPDH-R	GAAGGCTGGGGCTCATTT
Hsa_circ_TRIM22-F	TGCTCCCACAGATTGCTCCA
Hsa_circ_TRIM22-R	AGGGAGCAGTGCAATGGATTT
